# Disabling a conserved module confers broad‐spectrum resistance

**DOI:** 10.1111/jipb.70286

**Published:** 2026-05-07

**Authors:** Zhen Zeng, Yachun Su, Hui Ling, Youxiong Que

**Affiliations:** ^1^ State Key Laboratory of Tropical Crop Breeding, Institute of Tropical Bioscience and Biotechnology, Sanya Research Institute Chinese Academy of Tropical Agricultural Sciences Sanya 572024 China; ^2^ Key Laboratory of Sugarcane Biology and Genetic Breeding, Ministry of Agriculture and Rural Affairs, National Engineering Research Center for Sugarcane, College of Agriculture Fujian Agriculture and Forestry University Fuzhou 350002 China; ^3^ Guangxi Key Laboratory of Agricultural Resources Chemistry and Biotechnology, College of Smart Agriculture Yulin Normal University Yulin 537000 China

## Abstract

This commentary reviews the discovery by Yuan et al. of a conserved susceptibility module where fungal Gas2 stabilizes host SnRK1β1A to suppress nuclear immunity in rice. It discusses the mechanism for broad‐spectrum resistance via genome editing and considers the essential balance between enhanced defense and associated agronomic trade‐offs.

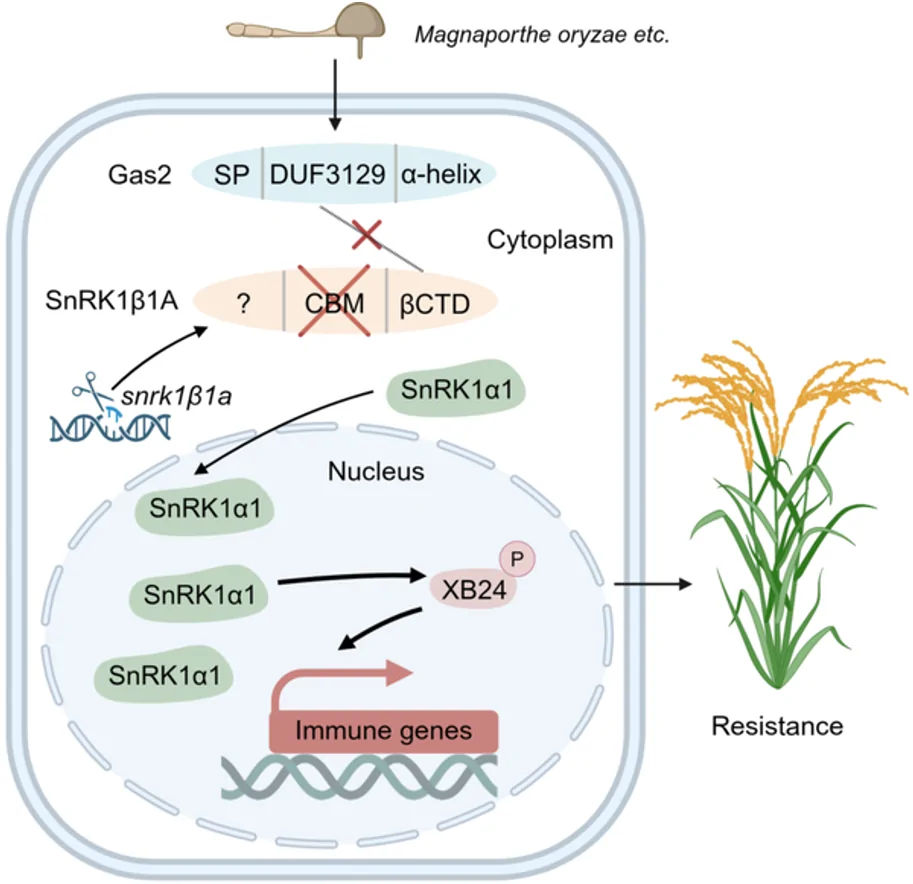

Crop losses caused by fungal diseases pose a persistent and severe threat to global food security (Savary et al., 2019). This challenge is particularly acute in rice, as diverse fungal pathogens infect the same host across distinct developmental stages and tissues, yet the resistance genes deployed in breeding are often race‐specific and easily overcome by rapidly evolving pathogen populations (Savary et al., 2019; Kusch et al., 2024). As a result, broad‐spectrum resistance has long been a central goal in crop protection, but the molecular logic (the causal framework linking conserved pathogen activities to a shared host regulatory node) underlying such resistance remains poorly defined. A key difficulty lies in the fact that most studies focus on one pathogen at a time, making it hard to identify the shared host vulnerabilities that are repeatedly exploited across pathosystems. A recent study addresses this problem in an elegant and conceptually important way. By starting from a conserved effector shared among six fungal pathogens, including *Magnaporthe oryzae*, *Rhizoctonia solani*, *Ustilaginoidea virens*, *Bipolaris oryzae*, *Fusarium fujikuroi*, and *F. graminearum*, the authors uncover a common virulence strategy and identify a host susceptibility module whose disruption confers broad‐spectrum fungal resistance in rice (Yuan et al., 2026).

A key strength of this study is that it extends beyond the description of a broad‐resistance phenotype to define a mechanistic cascade linking fungal invasion to host susceptibility. The authors identify Gas2 as a conserved fungal protein harboring a DUF3129 domain and a C‐terminal α‐helical region essential for virulence. They further demonstrate that Gas2's orthologs from divergent rice fungal pathogens function as conserved effectors that can be translocated into host cells to promote disease (Yuan et al., 2026). This striking conservation across taxonomically distinct pathogens strongly suggests that Gas2 targets a host process of broad importance for fungal susceptibility. That said, the most systematic evidence for the intracellular effector‐like behavior of Gas2 came from *M. oryzae*. The other five pathogens were validated primarily through genetic complementation and virulence analyses, without equivalent examination of their translocation or subcellular trafficking. Consequently, whether all Gas2 orthologs share identical host‐entry mechanisms remains an open question.

As summarized in Figure 1, the secreted Gas2 is translocated into rice cells and directly interacts with the βCTD domain of SnRK1β1A, a regulatory subunit of the SnRK1 energy‐sensing kinase complex. Crucially, Gas2 suppresses K267‐dependent ubiquitination of SnRK1β1A, thereby preventing its proteasomal degradation (Yuan et al., 2026). It remains unknown whether K267 is conserved in SnRK1β homologs of other crops, and which E3 ligase mediates its ubiquitination. Notably, the K267R substitution stabilizes SnRK1β1A and would therefore be expected to enhance susceptibility rather than confer resistance. Thus, these pathogens enhance virulence not by degrading host defense components but by stabilizing the host factor SnRK1β1A, which actively suppresses immunity.

**Figure 1:**
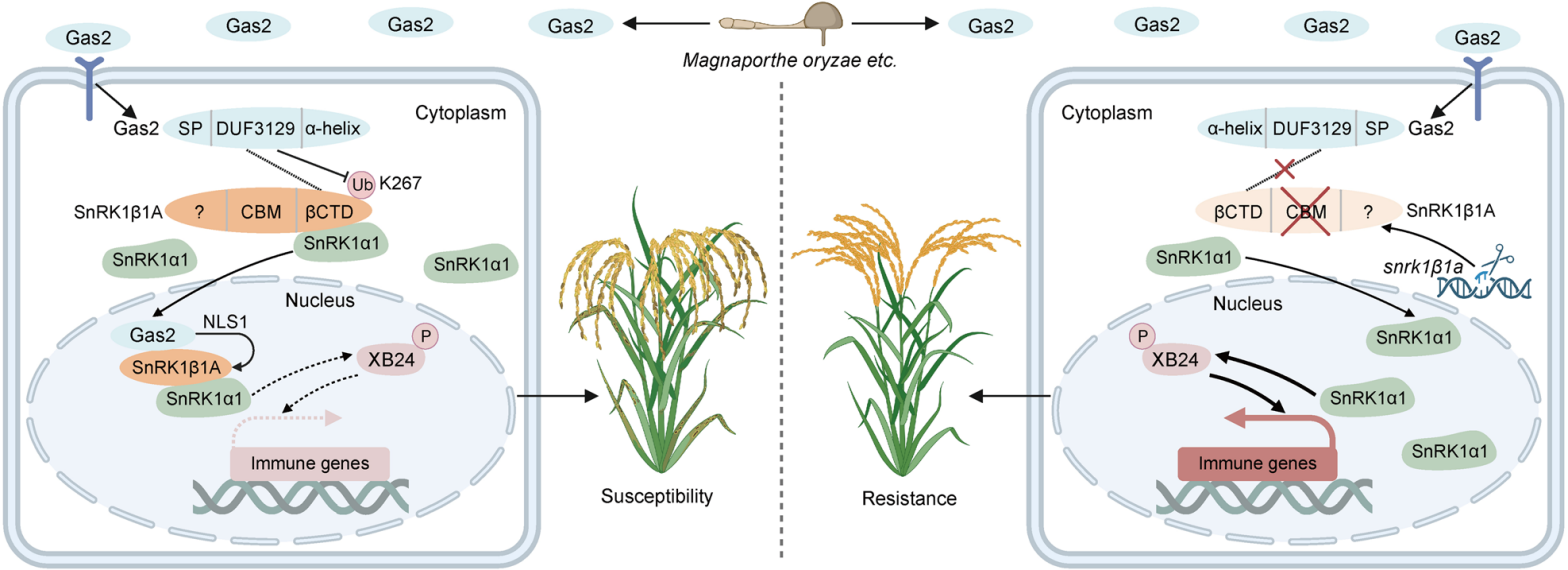
A conserved fungal Gas2‐SnRK1β1A module orchestrates broad‐spectrum fungal resistance in rice Left panel (Susceptible state): Multiple taxonomically distinct rice fungal pathogens (e.g., *Magnaporthe oryzae*) secrete conserved Gas2 effectors. Gas2 harbors an N‐terminal signal peptide (SP) for secretion, a DUF3129 domain containing a functional nuclear localization signal (NLS1), and a C‐terminal α‐helix essential for virulence. After translocation into rice cells, Gas2 directly interacts with the βCTD domain of SnRK1β1A, a regulatory subunit of the SnRK1 energy‐sensing kinase complex. This interaction suppresses K267‐dependent ubiquitination (Ub), preventing its 26S proteasome‐mediated degradation and stabilizing the protein. Concurrently, Gas2 drives the nuclear accumulation of SnRK1β1A via its NLS1 motif. In the nucleus, SnRK1β1A inhibits SnRK1α1 by suppressing its kinase activity and limiting its nuclear accumulation, thereby reducing phosphorylation of the immune substrate XB24 and dampening defense‐related gene expression, ultimately facilitating fungal colonization and disease symptoms (e.g., grain discoloration). Right panel (Resistant state): CRISPR‐Cas9‐mediated knockout of *SnRK1β1A* disrupts the virulence module. In the absence of SnRK1β1A, SnRK1α1 is released from inhibition, accumulates in the nucleus, and phosphorylates XB24. This triggers robust activation of immune gene expression, conferring broad‐spectrum resistance to all tested fungal pathogens. The *snrk1β1a* mutants exhibit normal growth and yield under field conditions but show earlier heading and increased sensitivity to high salinity, highlighting both the potential and trade‐offs of targeting this module for disease resistance breeding.

SnRK1 is well‐established as a central energy‐sensing kinase complex governing stress adaptation and defense regulation (Xu et al., 2025). However, the specific functions of individual β subunits in broad‐spectrum fungal susceptibility have remained largely elusive (Yang et al., 2022). This study identifies SnRK1β1A as the critical host susceptibility factor hijacked by diverse fungal pathogens during infection, and further integrates this protein stabilization event into a nuclear immune regulatory pathway.

Notably, Gas2 both increases SnRK1β1A protein abundance and promotes its nuclear accumulation, a relocalization that depends on the NLS1 motif of Gas2 and its physical interaction with SnRK1β1A. Once enriched in the nucleus, SnRK1β1A acts as a potent negative regulator of SnRK1α1‐mediated immunity by inhibiting the kinase activity of SnRK1α1. The data support a model in which Gas2‐driven nuclear accumulation of SnRK1β1A suppresses SnRK1α1‐dependent phosphorylation of the immune substrate XB24, thereby dampening downstream defense gene expression (Figure 1; Yuan et al., 2026). In this way, Gas2 coordinates protein stabilization, relocalization, and immune suppression into a unified virulence cascade.

This mechanism is particularly insightful because it explains how taxonomically distinct fungal pathogens converge to cause disease via a shared host‐susceptibility pathway. In the susceptible state (Figure 1, left panel), conserved Gas2 effectors sustain high nuclear levels of SnRK1β1A, leading to blunted immune gene activation and enhanced disease progression (Yuan et al., 2026). The study thus identifies not merely a virulence factor or a host target, but a unified susceptibility module that links conserved fungal effectors to nuclear repression of host immunity.

Mechanistically, the resistant state is equally well‐defined. When *SnRK1β1A* is genetically disrupted, Gas2 can no longer assemble the nuclear immune‐suppressive module. SnRK1α1 is released from inhibition, XB24 phosphorylation is restored, and robust defense gene activation occurs (Figure 1, right panel; Yuan et al., 2026). Interestingly, this molecular shift confers enhanced resistance to all six tested fungal pathogens. In breeding, such broad‐spectrum efficacy is particularly notable, as it differs fundamentally from most resistance genes, which rely on race‐specific effector recognition and are prone to rapid resistance breakdown in the field. Conceptually, the Gas2‐SnRK1β1A mechanism belongs to the loss‐of‐susceptibility category, more closely resembling *mlo*‐based resistance than classical NLR‐mediated race‐specific immunity. It differs from balanced broad‐spectrum systems such as the *Pigm* locus in rice, which achieves resistance through a competitive NLR architecture designed to minimize yield cost.

The study also clarifies why broad‐spectrum resistance mechanisms are rarely uncovered by conventional single‐pathogen studies. When each pathosystem is studied separately, Gas2 might appear to be just another virulence‐associated protein, and SnRK1β1A just another context‐dependent host regulator (Yuan et al., 2026). Only in a comparative multi‐pathogen framework does the convergent evolutionary logic of this interaction become clear: Divergent fungal pathogens retain homologous effector‐like proteins, all of which target the same host factor to suppress core immune outputs (Seong and Krasileva, 2023). This evolutionary convergence is what endows the mechanism with both profound biological significance and high translational breeding value. The convergent stabilization of a negative immune regulator may reflect an evolutionarily stealthy strategy across taxonomically distant fungal pathogens. Nevertheless, this convergence does not guarantee durable resistance, as pathogens could either alter Gas2 interfaces or deploy unrelated effectors to suppress the same host pathway.

The implications extend far beyond the rice–fungus pathosystem. A promising direction for future research is to apply population genetics and comparative genomics to pathogen populations to identify effectors or effector domains highly conserved within and across fungal species that infect the same crop (Liu et al., 2023; De la Concepcion et al., 2024). Such molecules likely mark host targets with persistent adaptive importance for pathogens. Gas2 serves as an informative example: Its conservation across six major rice fungal pathogens directly led to the discovery of a host regulatory node with broad pathological relevance. Whether other fungal pathogens also employ Gas2‐like effectors to target SnRK1β homologs remains an open question.

A parallel and complementary strategy starts from the host side. Conserved functional domains across plant species, combined with resistance/susceptibility alleles identified via intraspecific population genetics, can uncover host regulatory nodes repeatedly linked to broad‐spectrum disease phenotypes (Wu et al., 2025). These host candidates can then be used to reverse‐search for their interacting pathogen effectors. Conversely, conserved pathogen effectors can be used to forward‐identify shared host susceptibility factors. Integrating these two complementary approaches will provide a powerful framework for dissecting the molecular basis of broad‐spectrum resistance in diverse crops.

More broadly, this work highlights a critical and underappreciated feature of susceptibility genes: Many susceptibility factors are not passive host liabilities, but infection‐inducible regulatory nodes that pathogens actively stabilize and relocalize to suppress immunity (Yuan et al., 2026). The identification of K267 as a critical ubiquitination site further refines the mechanistic basis of Gas2‐mediated stabilization, although how this knowledge can be best translated into engineering strategies remains to be determined. Host factors of this type are especially desirable breeding targets, as they represent pathogen‐sensitive regulatory checkpoints rather than irreplaceable structural components required for plant growth (Li et al., 2022). In this study, *snrk1β1a* mutants exhibited normal growth and yield under standard field conditions; however, they displayed an earlier heading date and increased sensitivity to high salinity in specific agroecological environments. Overall, the trade‐off profile is more moderate than, but conceptually comparable to, that seen for *mlo*‐based resistance. In the model summarized in Figure 1, diverse fungal pathogens deploy a conserved intracellular effector to reinforce a nuclear susceptibility module, whereas genetic disruption of the host target SnRK1β1A shifts the balance toward SnRK1α1‐dependent immune activation and broad‐spectrum disease resistance.

Taken together, Yuan et al. (2026) provide both strong mechanistic clarity and a valuable foundation for crop resistance breeding. Their work uncovers a convergent fungal virulence strategy centered on the conserved effector Gas2, defines SnRK1β1A as a key host susceptibility factor stabilized by Gas2 through inhibition of K267 ubiquitination, and links Gas2‐mediated nuclear accumulation to the repression of XB24 phosphorylation and immune gene activation. For rice breeding, these findings position SnRK1β1A as a compelling candidate target for engineering broad‐spectrum resistance. Several questions remain: Whether SnRK1β homologs in other crops are similarly targeted by Gas2‐like effectors, how the K267 site can be leveraged in genome editing without incurring unacceptable agronomic costs, and why the observed convergence can be translated into durable field resistance or prove vulnerable to pathogen counter‐adaptation. Addressing these concerns will determine how broadly this paradigm can be extended across crop systems.

## CONFLICTS OF INTEREST

The authors declare no conflicts of interest.

## AUTHOR CONTRIBUTIONS

Q.Y. conceptualized the commentary, Z.Z. S.Y. and L.H. wrote the manuscript, and Z.Z. prepared the schematic figure. All authors reviewed and approved the final version.

## References

[jipb70286-bib-0001] De la Concepcion, J.C. , Langner, T. , Fujisaki, K. , Yan, X. , Were, V. , Lam, A. , Saado, I. , Brabham, H. , Win, J. , Yoshida, K. , et al. (2024). Zinc‐finger (ZiF) fold secreted effectors form a functionally diverse family across lineages of the blast fungus *Magnaporthe oryzae* . PLoS Pathog. 20: e1012277. 10.1371/journal.ppat.1012277 PMC11213319 38885263

[jipb70286-bib-0002] Kusch, S. , Frantzeskakis, L. , Lassen, B. , Kümmel, F. , Pesch, L. , Barsoum, M. , Walden, K , and Panstruga, R. (2024). A fungal plant pathogen overcomes *mlo*‐mediated broad‐spectrum disease resistance by rapid gene loss. New Phytol. 244: 962–979. 10.1111/nph.20063 39155769

[jipb70286-bib-0003] Li, S. , Lin, D. , Zhang, Y. , Deng, M. , Chen, Y. , Lv, B. , Li, B. , Lei, Y. , Wang, Y. , Zhao, L. , et al. (2022). Genome‐edited powdery mildew resistance in wheat without growth penalties. Nature 602: 455–460. 10.1038/s41586-022-04395-9 35140403

[jipb70286-bib-0004] Liu, J. , Nie, B. , Yu, B. , Xu, F. , Zhang, Q. , Wang, Y. , and Xu, W. (2023). Rice ubiquitin‐conjugating enzyme OsUbc13 negatively regulates immunity against pathogens by enhancing the activity of OsSnRK1a. Plant. Biotechnol. J. 21: 1590–1610. 10.1111/pbi.14059 PMC10363768 37102249

[jipb70286-bib-0005] Savary, S. , Willocquet, L. , Pethybridge, S. , Esker, P. , McRoberts, N. , and Nelson, A. (2019). The global burden of pathogens and pests on major food crops. Nat. Ecol. Evol. 3: 430–439. 10.1038/s41559-018-0793-y 30718852

[jipb70286-bib-0006] Seong, K. , and Krasileva, K. (2023). Prediction of effector protein structures from fungal phytopathogens enables evolutionary analyses. Nat. Microbiol. 8: 174–187. 10.1038/s41564-022-01287-6 PMC9816061 36604508

[jipb70286-bib-0007] Wu, Y. , Zhao, C.J. , Zhang, Y. , Shen, C. , Zhang, Y. , Zhang, X. , Gao, L. , Zeng, L. , Ke, Q. , Qin, L. , et al. (2025). Inactivation of *β‐1,3‐glucan synthase‐like 5* confers broad‐spectrum resistance to *Plasmodiophora brassicae* pathotypes in cruciferous plants. Nat. Genet. 57: 2302–2312. 10.1038/s41588-025-02306-y PMC12425816 40890362

[jipb70286-bib-0008] Xu, Q. , Kong, F. , and Yang, W. (2025). SnRK1 as the core node integrating energy homoeostasis, stress adaptation and hormonal crosstalk in plants. Plant Cell Environ. 48: 7830–7847. 10.1111/pce.70074 40692523

[jipb70286-bib-0009] Yang, J. , Zhang, N. , Wang, J. , Fang, A. , Fan, J. , Li, D. , Li, Y. , Wang, S. , Cui, F. , Yu, J. , et al. (2022). SnRK1A‐mediated phosphorylation of a cytosolic ATPase positively regulates rice innate immunity and is inhibited by *Ustilaginoidea* virens effector SCRE1. New Phytol. 236: 1422–1440. 10.1111/nph.18460 36068953

[jipb70286-bib-0010] Yuan, G. , Lu, X. , Wang, X. , Li, M. , Wang, S. , Huang, Z. , Li, Z. , Zhang, F. , Zhang, X. , Yang, J. , et al. (2026). Inactivating *SnRK1β1A* promotes broad‐spectrum disease resistance in rice. Nature 653: 474–482. 10.1038/s41586-026-10273-5 41882350

